# Mature neural responses to Infant-Directed Speech but not Adult-Directed Speech in Pre-Verbal Infants

**DOI:** 10.1038/srep34273

**Published:** 2016-09-28

**Authors:** Varghese Peter, Marina Kalashnikova, Aimee Santos, Denis Burnham

**Affiliations:** 1 MARCS Institute for Brain, Behaviour and Development, Western Sydney University, Penrith, Australia

## Abstract

Infant directed speech (IDS), the speech register adults use when talking to infants, has been shown to have positive effects on attracting infants’ attention, language learning, and emotional communication. Here event related potentials (ERPs) are used to investigate the neural coding of IDS and ADS (adult directed speech) as well as their discrimination by both infants and adults. Two instances of the vowel /i/, one extracted from ADS and one from IDS, were presented to 9-month-old infants and adults in two oddball conditions: ADS standard/IDS deviant and IDS standard/ADS deviant. In Experiment 1 with adults, the obligatory ERPs that code acoustic information were different for ADS and IDS; and discrimination, indexed by mismatch negativity (MMN) responses, showed that IDS and ADS deviants were discriminated equally well; although, the P3a response was larger for IDS suggesting it captured adults’ attention more than did ADS. In infants the obligatory responses did not differ for IDS and ADS, but for discrimination, while IDS deviants generated both a slow-positive mismatch response (MMR) as well as an adult-like MMN, the ADS deviants generated only an MMR. The presence of a mature adult-like MMN suggests that the IDS stimulus is easier to discriminate for infants.

Adults use a special speech register known as infant directed speech (IDS) when addressing young infants. Compared to adult directed speech (ADS), IDS is characterised by speakers’ exaggerated facial expressions (e.g., raised eyebrows, widened eyes, smiles)^1^,^2^, simplified grammar^3^, slower tempo, higher pitch and greater pitch range, greater affect, and hyperarticulated vowels^4^,^5^. In addition to contributing to infants’ early social-emotional development^6^, IDS has also been proposed to facilitate the process of language acquisition in the first years of life^7^, although the mechanisms via which IDS might do so are still to be defined^8^. The studies reported here investigated the neural bases for IDS and ADS processing in adults and young infants to determine whether processing advantages for IDS over ADS can be observed in the first year of life, and whether any such advantages are also evident in mature language users.

Vowel hyperarticulation, a speaker’s tendency to exaggerate the articulation of vowels in their speech^9^, has been proposed to serve a didactic function in IDS. Vowel hyperarticulation is indexed by measuring the area of the vowel triangle that results from plotting F1 and F2 (1^st^ and 2^nd^ formant) values for the corner vowels (/i/, /u/, /a/) in a two-dimensional F1/F2 vowel space for a particular speech register, e.g., IDS, and comparing the resultant area with a control, e.g., ADS. Kuhl *et al.*^5^ compared IDS and ADS vowel triangles produced by mothers speaking English, Russian, and Swedish, and found vowel hyperarticulation in all three language groups. This has been shown to have linguistic benefits; mothers whose IDS shows greater vowel hyperarticulation have infants who show higher performance in speech discrimination tasks^10^ and lexical processing^11^. Vowel hyperarticulation has also been observed in other registers, but only where speakers address listeners who have a particular perceived linguistic capacity such as in speech to foreigners^12^, computers^13^, parrots^14^, but not in speech to cats and dogs^4^. Vowel hyperarticulation is also absent in IDS to infants with a hearing impairment^15^,^16^ further indicating that mothers may unconsciously adapt the linguistic characteristics of their speech according to the sensory and/or linguistic needs of their infant.

The acoustic and affective components of IDS have also been demonstrated to vary according to the infants’ age and the communicative interaction between the mother and her infant. For example, adults’ blind ratings of the communicative intent of low-pass filtered IDS addressed to newborns, three-, six-, nine-, and 12-month-olds, indicate that mothers’ speech is high on the dimension of ‘comfort and soothe’ to newborns, ‘encourage attention’ to three- and 12-month-olds, ‘express affection’ to six-month-olds, and ‘direct behaviour’ to nine-month-olds. In addition, mothers also modify the pitch characteristics of their speech according to infant age^17^, infant gender^18^, and feedback from the infant during their interaction^19^. Smith and Trainor^19^ used a double-video set up, in which the mother and infant sat in different rooms, and mothers could see and hear their infants on a computer monitor. Mothers were told that their infant could hear and see them, but in reality, the infant saw an experimenter who acted in a manner that was either congruent or incongruent with the emotional content in the mother’s speech. When the experimenter acted incongruently, mothers systematically increased their pitch suggesting that they were responding to cues from their infants’ behaviour in the course of the interaction.

Therefore, the qualities of IDS appear to be shaped by reciprocal interaction between infants and their adult interlocutors. In accord with such a notion, robust behavioural evidence indicates that infants both discriminate IDS from ADS *and* show an early preference for IDS^20^,^21^,^22^,^23^. Such behavioural data are backed up by neurophysiological evidence, which indicates that young infants indeed respond differentially to ADS and IDS. Increased activation for IDS over ADS in frontal brain regions has been found using near infrared spectroscopy with newborns^24^ and 4- to 13-month-old infants^25^. Santesso *et al.*^26^ also reported increased electroencephalogram (EEG) power at frontal sites when 9-month-old infants listened to IDS compared with ADS. This increased neural activity in the frontal regions in response to speech can lead to higher attention and more successful encoding of the incoming speech stream, which are highly beneficial for language learning.

The neural processing of specific acoustic and linguistic information in IDS can be studied with precise time-resolution using event related potentials (ERPs). An auditory ERP is the average pattern of electrical activity generated by large groups of brain cells in response to a sound stimulus and are thought to reflect sound detection, complexity and feature extraction processes^27^,^28^. Changes to auditory ERPs occur across age due to neural maturation^29^. For example, below the age of 12 months, two ERP peaks are observed: P150 (positive peak at approximately 150 ms from stimulus onset) and N250 (negative peak at approximately 250 ms)^30^,^31^. In contrast, auditory ERPs in adults comprise two negative peaks N1 (90–110 ms) and N2 (220–280 ms) as well as two positive peaks P1 (40–60 ms) and P2 (140–170 ms)^29^,^32^. These peaks in the auditory ERPs are often called “obligatory responses” as they are generated by almost all audible sounds.

Two studies have investigated ADS and IDS processing in infants using obligatory ERPs. Zangl and Mills^33^ recorded ERPs from 6- and 13-month-old infants while they listened to familiar or unfamiliar words in either ADS or IDS. There was a larger response to IDS for familiar words than ADS over the left hemisphere between 600–800 ms from word onset in six-month-old infants. In contrast, 13-month-old infants generated a larger response between 200–400 ms for familiar words presented in IDS as well as between 600–800 ms for both familiar and unfamiliar words in IDS across hemispheres as compared to ADS. The increased activity in the 600–800 ms time window for IDS was attributed to increased attention to and arousal by IDS stimuli as compared to ADS stimuli. More recently, Zhang *et al.*^34^ investigated young infants’ neural responses to vowels, which were formant-exaggerated in ordered to mimic the vowel hyperarticulation in IDS. They presented six- to 12-month-old infants with formant-exaggerated and non-exaggerated forms of the synthetic vowel /i/ in separate blocks and found larger P150 and N250 responses to formant-exaggerated vowels than to non-exaggerated vowels. Source localisation also revealed a bilateral temporal-parietal-frontal cortical network that was sensitive to formant exaggeration. Zhang *et al.* therefore hypothesised that activation of this network facilitates language learning via cortical interactions in the perceptual-motor system.

While obligatory responses provide important information about the processing of spectral and temporal cues in the stimulus, they do not provide any information about stimulus discrimination. Auditory discrimination, for infants and adults, is an important determinant of language learning outcomes^35^,^36^. Specifically for young infants, discrimination between ADS and IDS is essential for the infant to identify the speech that is directed at them^37^, and which, as has been shown above, contains information that facilitates language development^10^,^11^. One ERP component which is used widely in speech discrimination research is mismatch negativity (MMN), which reflects early stages of change detection in the auditory system. In the MMN paradigm an infrequent stimulus (deviant) is presented among a series of repeatedly presented stimuli (standards). The MMN response is the result of a pre-attentive memory based comparison process in which each incoming sound is compared with the memory trace formed by the preceding sounds. If the features of the incoming sound do not match the memory trace, an MMN response results^38^,^39^. The MMN is represented by a negative peak (between 100–250 ms from stimulus onset in adults) in the difference waveform between the ERPs to deviants and ERPs to the standards. In adults, the MMN response is sometimes followed by a positive response between 200–300 ms at the fronto-central electrodes. This response, called P3a, reflects the involuntary attention shift to the deviant stimuli^40^. In newborns and young infants the MMN is seen as a broad positive response (instead of a negative peak) and is commonly referred to as an MMR (mismatch response)^41^,^42^. This positive MMR changes to the more adult-like negative MMN within two years of age for most auditory contrasts^43^,^44^. The factors that determine the polarity of the mismatch response in infants include neural maturation (the proportion of infants showing positive MMR decreases and proportion of infants with negative MMN increases between two-six months of age)^45^, and deviance magnitude (large deviants elicit adult-like MMN whereas small deviants elicits positive MMR in two- to seven-month-old infants)^43^.

Here two experiments are reported on the discrimination of an isolated speech sound (vowel /i/) produced in IDS and ADS by two groups of participants: adults (Experiment 1) and nine-month-old infants (Experiment 2). We employed an MMN paradigm in which /i/ vowels extracted from our recordings of naturally produced IDS and ADS (as opposed to synthetic stimuli)^34^ were used as the standard and deviant stimuli. Our aim was twofold: (1) to assess the effect of IDS and ADS on the obligatory ERP response (N1-P2 response in adults and P150 -N250 response in infants) using isolated vowel stimuli; and (2) to assess how IDS and ADS deviants affect the ERP discriminatory response (MMN/MMR) in adults and infants. With respect to Experiment 1, research on adult neural processing of IDS is scarce, but it has been suggested that while mothers of pre-verbal infants show increased cortical activation to IDS, other adults do not^46^. More specifically it has been found that individual features of IDS such as heightened pitch^47^,^48^ and vowel hyperarticulation^49^ result in larger MMN responses in adults. The adult experiment here was conducted to investigate adult responses to natural IDS stimuli in an MMN paradigm and to provide a basis for comparison with Experiment 2 with infants. In Experiment 2 IDS/ADS discrimination was assessed in nine-month-old infants, an age at which infants are in the process of attuning to the phonological inventory of their native language^50^. We used a narrower age range for infant participants than in previous ERP studies^34^ as the characteristics of IDS^17^ and infants’ preferences for this vary considerably across the first year of life^51^,^52^, so brain responses to IDS may also vary across age. We predicted that there will be differences in infants’ obligatory responses to IDS vs. ADS due to acoustic cue differences between these registers^33^,^34^. Since infants prefer listening to IDS^20^,^21^,^22^,^23^ and behaviourally discriminate speech better in IDS^53^, we also predicted that IDS deviants would elicit larger and more mature MMN/MMR in infants than would ADS deviants. All the experimental methods used in the study were approved by the ethics committee for human research at Western Sydney University (approval number: H9660). The methods were carried out in accordance with approved guidelines. Informed consent was obtained from all the adult participants (Experiment 1) and parents of infant participants (Experiment 2).

## Experiment 1

### Method

#### Participants

Twenty one adults between 18 and 40 years of age (14 females, *M* age: 28.67 years, SD: 10.84 years) participated. All were native English speakers and reported no hearing difficulties. No participant was a parent of a pre-verbal infant. Data from one participant was excluded due to technical error, with a final sample of 20.

#### Stimuli

IDS and ADS stimuli were selected from an existing corpus of audio recordings of mothers interacting with their nine-month-old infants (IDS) and an adult experimenter (ADS). In these interactions, mothers were instructed to use the target words ‘sheep’, ‘shoe’ and ‘shark’ to elicit the three corner vowels /i/, /u/, and /a/. One particular mother’s IDS and ADS was selected for present purposes following an inspection of the acoustic, affective, and linguistic features of this mother’s speech in the two registers, which showed that, as expected, she produced longer vowels, higher pitch and greater pitch range, greater affect, and a greater vowel triangle in IDS than in ADS (Table 1 and Supplementary material). For present purposes, one exemplar of the vowel /i/ was selected for each register matching the IDS and ADS vowel in duration. As a check, nine adult native speakers of Australian English were asked to identify the vowel in an open-ended question, and to report whether the speaker was addressing an infant (IDS) or an adult (ADS). The selected /i/ exemplars in ADS and IDS were rated high (>80%) in terms of vowel identity and speech register.

#### Design

EEG was recorded in two oddball conditions: ADS deviant and IDS deviant. In the ADS deviant condition, the IDS stimulus was presented as the standard and ADS stimulus as the deviant, and the opposite was the case in the IDS deviant condition. In each condition 80% of the stimuli were standards and 20% deviants. There were a total of 1200 stimuli in each condition (960 standards, 240 deviants). Each condition was divided into two blocks of 600 stimuli each, with the same 80/20 standard/deviant proportion. Each block began with 10 repetitions of the standard stimulus following which standards and deviants were presented in a pseudo-random order with a minimum of two and a maximum of eight standards between the deviants. The inter-stimulus interval (sound offset to next sound onset) was 500 ms. The order of blocks was counterbalanced across participants. Stimulus delivery was controlled using Presentation 16.3 (Neurobehavioral Systems) running on a PC.

#### EEG recording

Participants sat 1 m from an LCD screen and watched a silent video of their choice with subtitles. They were instructed to ignore the sounds they heard and concentrate on the video. Their continuous EEG was recorded using 129 channel Hydrocel Geodesic Sensor Net (HCGSN), NetAmps 300 amplifier and NetStation 4.5.7 software (EGI Inc) at a sampling rate of 1000 Hz with the reference electrode placed at Cz. The electrode impedances were kept below 50 kΩ. The continuous EEG was saved for offline analysis.

#### Offline analysis

The EEG data were analysed offline using the fieldtrip toolbox^54^ in MATLAB2014a (Natick, MA, USA). Portions of EEG containing large artefacts were visually identified and removed. The continuous EEG was then band pass filtered using Butterworth infinite impulse response (IIR) filter between 0.1–20 Hz, and then divided into epochs from −100 to 400 ms relative to sound onset. Baseline activity, defined as the mean amplitude between −100 to 0 ms was subtracted from each epoch. Noisy EEG channels were interpolated by averaging the neighbouring electrodes weighted by distance (average: 3 channels/subject, range 0–9). Trials with amplitude exceeding ± 100 μV were rejected. The epochs were then digitally re-referenced to the common average reference. Each participant had at least 80% accepted trials for each standard and deviant type (ADS deviant M = 92.23%, SD = 7.86%; IDS deviant M = 92.31%, SD = 7.86%; *t* (19) = 0.07; p > 0.05). The epochs were averaged separately for standards and deviants (excluding the first 10 standards in each block and the standards that immediately followed a deviant) to obtain 4 ERP waveforms per participant (ADS Standard, IDS Deviant, IDS Standard, ADS Deviant). Difference waves were calculated by subtracting the ERPs to the same stimulus when it was presented as standard in one block from when it was presented as deviant in another block (IDS deviant-IDS standard, ADS deviant-ADS standard). The waveforms from individual subjects were averaged to create grand averaged waveforms.

#### Statistical analysis

The standard and deviant waveforms for the same stimulus (IDS deviant vs IDS standard; ADS deviant vs ADS standard) as well as the standard waveforms for IDS and ADS were subjected to separate non-parametric cluster-based permutation tests^55^ to identify whether the waveforms differed significantly at any particular time point. This data-driven analysis included all the electrodes except the two facial electrodes and all time points between 0 and 400 ms. A series of t-tests was computed at every electrode and every time point. From this analysis, clusters of electrodes and time points in which the response significantly differed from zero were identified. These clusters were formed over space by grouping electrodes (at least 3 adjacent electrodes) that had significant initial t-tests (p < 0.05, two-tailed) at the same time point. A permutation approach was used to control Type I error, involving comparing the clusters identified in the first step by randomly assigning conditions and repeating the multiple t-tests (1000 iterations). If the difference is real, then t-tests comparing randomly permuted conditions should yield no significant results. A cluster is considered significant if the *p* value in the cluster statistics is less than 0.05, i.e., less than 50 of the random permutations are significant.

**Figure 1:**
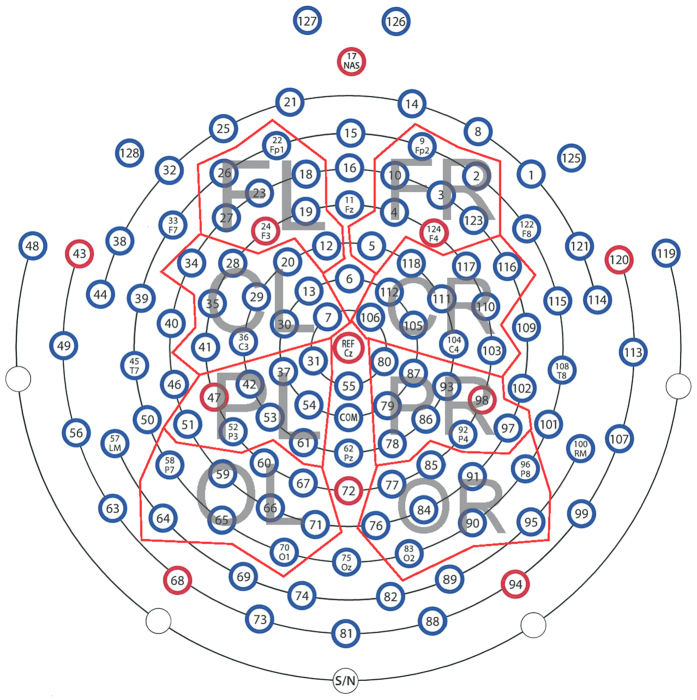
Electrode groupings used for the analysis. FL-frontal left, FR-frontal right, CL-central left, CR-central right, PL-parietal left, PR-parietal right, OL-occipital left, OR-occipital right.

The cluster-based permutation tests revealed two time windows where the standard and deviant waveforms differed for both ADS and IDS conditions (see Results and Discussion). Timing and polarity of the earlier time window resembled the MMN and the later time window resembled the P3a response. Given that the cluster-based permutation tests do not account for interactions between conditions, we performed further analysis using analysis of variance (ANOVA) to evaluate the difference between ADS and IDS conditions for MMN and P3a separately. The MMN and P3a amplitudes were computed from individual subjects as the mean amplitude in a uniform 50 ms time window that was centred at the peak latency of MMN and P3a in the grand averaged waveform for 72 electrodes. The electrodes were divided into 8 groups: frontal left (8 electrodes), frontal right (8 electrodes), central left (electrodes 10 electrodes), central right (10 electrodes), parietal left (9 electrodes), parietal right (9 electrodes), occipital left (9 electrodes) and occipital right (9 electrodes; Fig. 1). Similar groupings of electrodes are commonly used for the analysis of MMN responses from infants and adults^56^,^57^,^58^. The MMN and P3a amplitudes were subjected to separate 3-way ANOVAs with the factors stimuli (IDS, ADS), hemisphere (right, left) and location (frontal, central, occipital). Whenever appropriate, Greenhouse-Geisser correction was applied to account for potential violation of sphericity. Partial η^2^ was calculated as a measure of effect size.

## Results and Discussion

### Obligatory response to IDS and ADS

**Figure 2:**
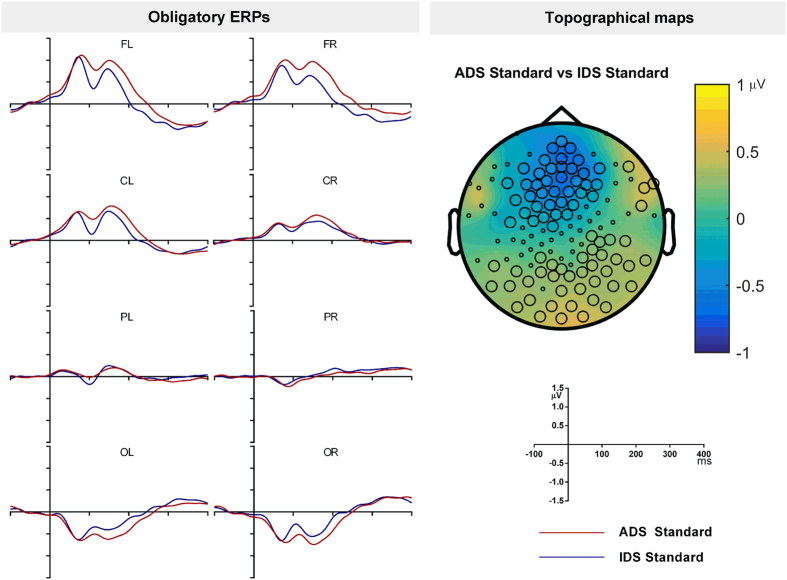
(**A**) The grand averaged ERP waveforms for the IDS and ADS standards from adult participants. (**B**) Topography of the difference between IDS and ADS at its peak. The highlighted electrodes belong to a statistically significant cluster.

**Figure 3:**
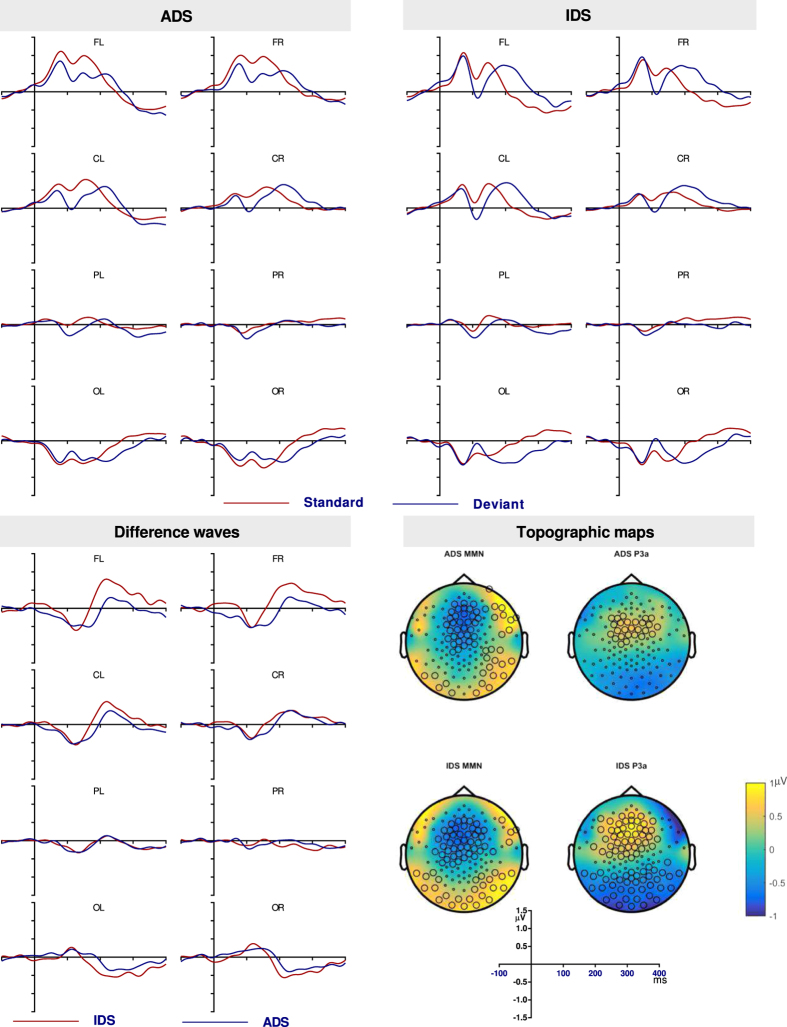
The standard and deviant waveforms for ADS (**A**), IDS (**B**) and the deviant minus standard difference waves (**C**) from adult participants. Topography of the deviant-standard wave at the MMN and P3a peaks (**D**). The highlighted electrodes belong to a statistically significant cluster.

The first aim was to assess the differences in ERPs to sound onset (N1-P2 response in adults) between IDS and ADS. Figure 2 shows the grand averaged ERPs to IDS and ADS presented as standards in different blocks. Statistical analysis by cluster based permutation test between IDS and ADS as standards revealed a significant negative cluster between 80 and 252 ms at frontal electrodes (p = 0.01) and a significant positive cluster between 78 and 277 ms at posterior electrodes (p = 0.003; Fig. 3).

The broad time windows where the obligatory response to IDS and ADS differed encompass the N1-P2 response range. The N1-P2 response is thought to reflect the processing of many of the spectral and temporal cues contained in speech that are critical for speech perception. Therefore the difference in ERP between ADS and IDS in the N1-P2 time range may well reflect the difference in spectral and temporal cues between ADS and IDS.

### Discriminatory responses for IDS vs ADS

The second aim was to assess the discriminability of IDS deviants from standards, and ADS deviants from standards. Standard, deviant and deviant minus standard difference waveforms are shown in Fig. 3. As can be seen, the difference waveforms showed an MMN between 100–200 ms and a P3a between 200–300 ms. The significance of these observations was confirmed by statistical analysis. The cluster-based permutation test on standard and deviant ERP waveforms for ADS revealed two positive clusters and one negative cluster (Table 2 and Fig. 3). The negative cluster in the frontal electrodes was in the MMN time range (67–192 ms) whereas the positive cluster in the frontal electrodes was in the P3a time range (207–272 ms). The analysis of the standard and deviant waveforms for IDS revealed two positive and two negative clusters. Similar to ADS, the negative cluster at frontal electrodes was in the MMN time range (76–166 ms) and the positive cluster was in the P3a time range (175–350 ms; Table 2 and Fig. 3).

ANOVA on MMN amplitude revealed a main effect of location F (1.20, 22.75) = 11.03, p = 0.002, partial η^2^ = 0.37. MMN at frontal (M = −0.45, SE = 0.11) and central (M = −0.33, SE = 0.07) locations were negative where the response at the occipital location was positive (M = 0.19, SE = 0.08). The posterior reversal of the polarity is indicative of the source of the activity in the auditory cortex^59^. No other main effects or interactions were significant (See Supplementary material for complete ANOVA results).

ANOVA of P3a amplitude revealed a main effect of stimulus F (1,19) = 13.34, p = 0.002, partial η^2^ = 0.41; IDS generated larger P3a responses (M = 0.21, SE = 0.03) compared to ADS (M = 0.08, SE = 0.03). The main effect of location was also significant F (1.32, 25.16) = 26.64, p = 0.001, partial η^2^ = 0.58. P3a at frontal (M = 0.47, SE = 0.09) and central (M = 0.39, SE = 0.06) locations were larger than at the occipital location (M = −0.43, SE = 0.09). The interaction between stimulus and location was also significant F (1.18, 22.45) = 7.53, p = 0.009, partial η^2^ = 0.28. A follow-up one-way ANOVA computed on each location revealed a main effect of stimulus at the frontal location F (1,19) = 13.90, p = 0.001, partial η^2^ = 0.42, but not the central and occipital locations.

In summary, the adults showed a difference between their obligatory responses to ADS and IDS. This finding is not surprising as adult N1-P2 obligatory responses have been shown to be sensitive to acoustic cues such as frequency^60^, rise time^61^, and VOT^62^. Thus the differences between IDS and ADS in their acoustic characteristics (fundamental frequency, formants, etc.) may well have led to the differences in their obligatory responses. However, adults in this experiment did *not* show any difference in MMN amplitude for IDS and ADS. It is possible that these adult participants would be more familiar with ADS than IDS, and the evidence on the effect of familiarity on MMN is mixed. Some studies have shown that familiar deviants presented along with unfamiliar standards elicit MMNs with higher amplitude^63^,^64^,^65^, but the opposite effect has been reported by others^66^,^67^, while other studies report no effect of stimulus familiarity on MMN^68^. The absence of a familiarity effect on MMN in adults suggests that the effect is dependent on the speech contrast being investigated. In this regard and turning to the P3a, both IDS and ADS elicited a P3a indicating that both deviants caused an involuntary attention shift or orienting response to the deviant sound^69^. However, it is of note that IDS generated a larger P3a than did ADS, indicating that the attention shift was larger for IDS. This suggests that, just as is the case for infants^20^,^21^,^22^,^23^, IDS is more attention-grabbing than ADS even for adults.

## Experiment 2

### Method

#### Participants

Twenty infants nine-month-old (11 females; *M* age: 9 month 16 days, *SD*: 10.24 days) participated. All, according to parental reports, were acquiring English as their first language, were born full-term, and were not at-risk for cognitive or language delay. Four infants were excluded as they did not render an adequate number of artifact free trials (at least 70 artifact-free deviant trials), so the final sample comprised 16 infants.

#### Stimuli and Design

Stimuli, design, and apparatus identical to Experiment 1 were used.

#### EEG recording

The infants sat on their parent’s lap approximately 1 m from an LCD screen and watched an age-appropriate silent video. Stimulus presentation and EEG recording was same as in Experiment 1.

#### Offline analysis

EEG analysis was performed using the fieldtrip toolbox^54^ running on MATLAB 2014a (Natick, MA, USA). Portions of EEG containing large artifacts were visually identified and removed. The EEG was then filtered using two band pass filter settings: 0.1 to 20 Hz and 3–18 Hz. This was done because the more common 0.1 to 20 Hz filter is most useful in identifying the positive MMR in infants whereas the 3–18 Hz filter will remove the low-frequency MMR response and allows visualisation of more adult-like negative MMNs^70^. This was the only difference in analysis from Experiment 1. After filtering, each subject had two sets of EEG traces which were then divided into epochs between −100 to 400 ms and baseline corrected between −100 to 0 ms. Noisy EEG channels were interpolated by averaging the neighbouring electrodes weighted by distance (average: 9 channels/subject, range 3–20). Trials with amplitude exceeding ± 100 μV were removed. Only those epochs that were accepted for both 0.1–20 Hz and 3–18 Hz filtering were selected. All the participants had at least 70 accepted deviant trials (ADS deviant M = 67.60%, SD = 19.15%; IDS deviant M = 63.88%, SD = 20.98%; *t* (15) = 0.65, p > 0.05). Deviant and standard epochs (excluding the first 10 epochs and standards that immediately follow a deviant) were averaged separately for each stimulus. Difference waves (ADS deviant-ADS standard; IDS deviant- IDS standard) were computed for every participant and grand averaged waveforms were computed.

#### Statistical analysis

Statistical analysis was performed separately for 0.1 to 20 Hz and 3 to 18 Hz filtered data. For the 0.1 to 20 Hz filtered data, cluster based permutation tests were performed to identify the time windows where the ADS standards and IDS standards differed significantly; which revealed no significant clusters (see Results and Discussion). A second cluster based permutation test was computed to identify the time windows where the standard and deviant waveforms differed significantly. This analysis revealed a broad positive cluster in the frontal electrodes for both IDS and ADS (see Results and Discussion). This was further analysed by computing averaged ERP amplitudes in three time windows; 100–200 ms; 200–300 ms; 300–400 ms. These were subjected to separate ANOVAs with the factors stimuli (IDS, ADS), hemisphere (right, left) and location (frontal, central, occipital). For the 3 to 18 Hz filtered data, cluster based permutation tests were performed only between standards and deviants, which revealed s significant cluster only for IDS.

## Results and Discussion

### 0.1–20 Hz filtered data

#### Obligatory response to ADS and IDS

**Figure 4:**
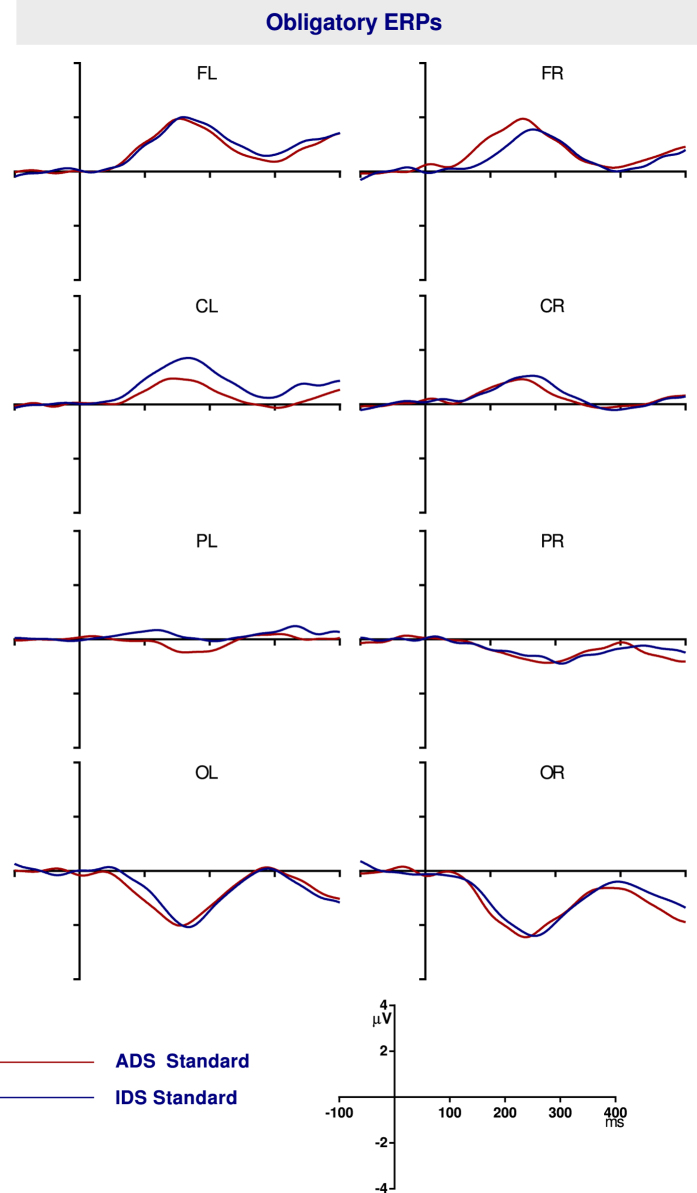
The ERP waveforms for the IDS and ADS standards in infants.

The ERPs to IDS and ADS when presented as standards are shown in Fig. 4. The ERPs show a positive P1 at around 150 ms and a negative N2 at around 250 ms. However cluster-based permutation statistics did not show any significant difference between the ERPs in the 0 to 400 ms range. Therefore statistically equivalent obligatory responses were obtained for ADS and IDS in infants.

#### Discriminatory response to ADS and IDS

**Figure 5:**
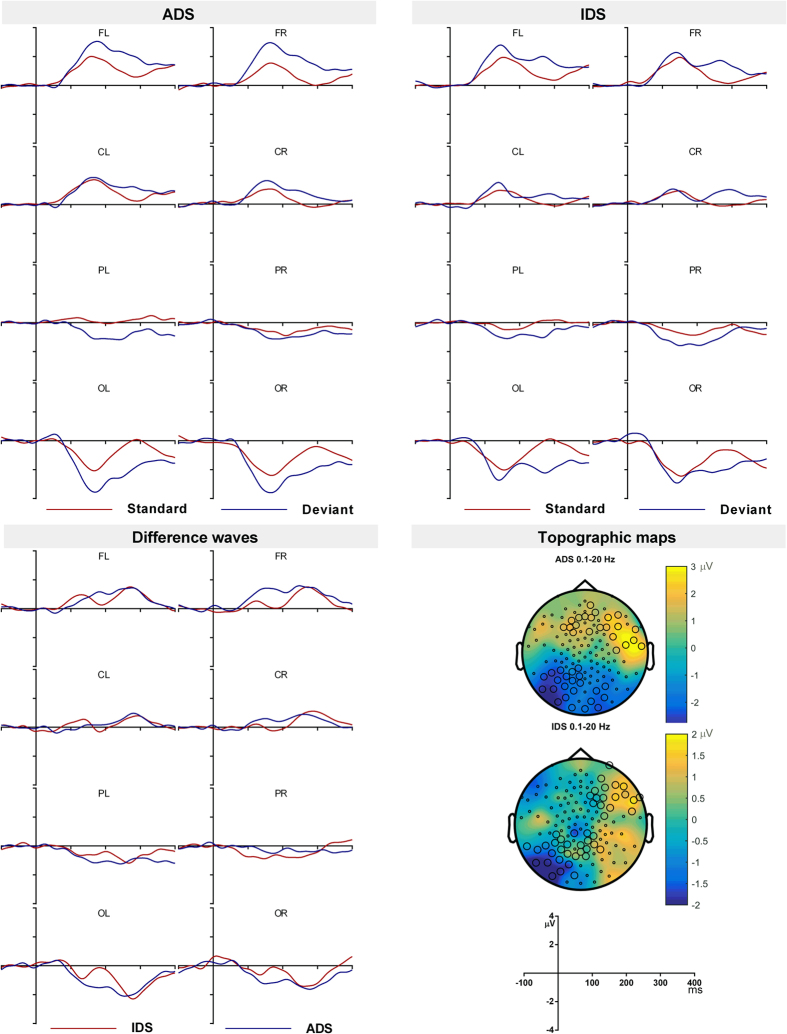
The standard and deviant waveforms for ADS (**A**), IDS (**B**) and the deviant minus standard difference waves (**C**) from infants in the 0.1–20 Hz filtered condition. The topographic maps of MMR at its peak (**D**). The highlighted electrodes belong to a statistically significant cluster.

The standard, deviant and deviant-standard difference waveforms for IDS and ADS are shown in Fig. 5. The difference waveform showed a broad positive peak at the frontal electrodes between 100 and 400 ms. Cluster-based permutation tests revealed that the positivity was significant between 98 and 380 ms for ADS and between 231 and 388 ms for IDS (Table 2) at the frontal electrodes, a broad positivity that is taken as the MMR response. There were also significant negative clusters at the posterior sites at approximately the above time windows. The posterior reversal of polarity suggests that the response has origins at the auditory cortex^59^. Figure 5 also shows the topography of the significant clusters.

MMR was further analysed by averaging the amplitude between 100 and 200 ms, 200 and 300 ms and 300 and 400 ms. A 3-way ANOVA computed on these time windows revealed a main effect of location between 100 and 200 ms F (1.34, 20.15) = 5.08, p = 0.027, partial η^2^ = 0.25 and 200 and 300 ms F (1.27, 19.02) = 11.38, p = 0.002, partial η^2^ = 0.43. Bonferroni corrected pairwise comparisons revealed that the response in the 200 to 300 ms time window at frontal and central locations was positive (frontal M = 1.05, SE = 0.34; central M = 0.57, SE = 0.27), which was significantly different from the negative occipital response (M = −1.35, SE = 0.36). Pairwise comparisons showed no significant differences across conditions in the 100 to 200 ms window. None of the other main effects or interactions were significant (see supplementary material for complete ANOVA results). Therefore the MMR response to ADS and to IDS did not differ in infants.

### 3–18 Hz filtered data

**Figure 6:**
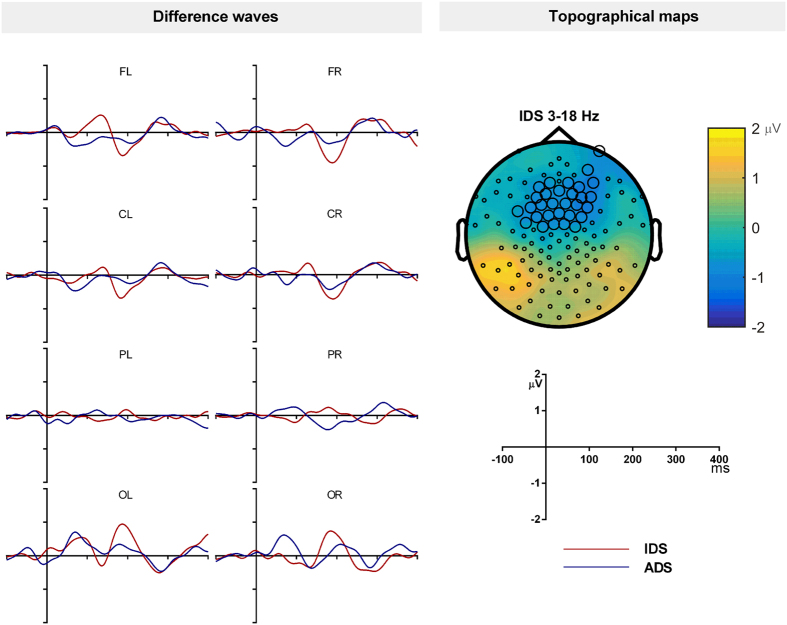
The deviant minus standard waveforms for ADS and IDS in infants in the 3–18 Hz filtered condition (**A**). The topographic maps of MMN at its peak (**B**). The highlighted electrodes belong to a statistically significant cluster.

The 3 to 18 Hz filtering was applied to remove the slow wave from the difference wave and therefore the cluster-based permutation test was not performed for the obligatory response. Figure 6 shows the difference waveforms for ADS and IDS. For IDS there was a difference waveform that showed a negative peak at the frontal electrodes. The cluster-based permutation tests on the standard and deviant waveforms showed a significant negative cluster at the frontal electrodes between 153 and 219 ms (Fig. 6; Table 2). Since the polarity and latency of this effect is similar to the adult MMN, this can be considered as the MMN response to IDS. The permutation tests on standards and deviants for ADS however did not show any significant clusters. Therefore only IDS stimuli generated adult-like MMN in infants when 3 to 18 Hz band pass filter was applied.

The results show no differences in obligatory responses were detected for IDS and ADS stimuli in our infant sample, but analyses of discriminatory responses demonstrated that a (positive) MMR and a (negative) MMN coexist in infants for IDS whereas only the MMR is present for ADS. While the functional significance of the MMR is still debated^31^,^70^, there is a general consensus that the MMR reflects less mature speech discrimination processes, which later develop into MMN. As for adults, the MMN in infants is thought to reflect the pre-attentive memory-based detection of the deviants^42^ whereas the MMR most likely reflects processes related to neural adaptation^42^ and differences in alertness or attention^31^,^71^. It is therefore argued that the MMN reflects cognitive aspects of change detection (which are more prevalent for easy to discriminate contrasts and in older infants) whereas MMR reflects precognitive aspects of change detection^71^. It is also reported that presence of MMN in infants for some contrasts is associated with better grammatical rule learning abilities^36^. It is therefore possible that MMN for IDS is related to the ability to learn grammatical rules from IDS.

## General Discussion

This study investigated the obligatory responses (N1-P2 in adults and P1-N1 in infants) and the discriminatory responses (MMN/MMR) to ADS and IDS in adults and infants. The results revealed differential response patterns depending on participants’ age and the register of the experimental stimuli. Adults had a more negative obligatory response to IDS in the N1-P2 latency range as compared to their response to ADS, but there were no differences in infants’ obligatory responses to ADS vs IDS. For discrimination, in adults, both ADS and IDS generated MMNs with similar amplitude; however, the adults’ P3a response was larger for IDS, indicating greater involuntary attention shift to IDS deviants. In contrast, in infants’ discrimination, when the appropriate narrow band pass filter (3 to 18 Hz) was employed an adult-like MMN was seen in infants’ responses to IDS but not to ADS.

Therefore our results indicate differential processing patterns of IDS and ADS in the adult and infant brain. Adults’ neural responses did not differ when they were presented with the task of detecting an ADS stimulus in a sequence of IDS stimuli or detecting an IDS stimulus in a sequence of ADS. Infants, however, only showed a mature discrimination response when presented with an IDS stimulus in a sequence of ADS stimuli but not vice versa. The transition of the mismatch response from positive to a negative wave is associated with more mature processing or processing of simpler stimuli^44^,^72^. Vowels present in IDS are distinguished from ADS based on exaggerated articulation (greater F1 and F2 values), greater pitch (F0), and higher rated affect, both in the stimuli here and in other studies of IDS and ADS^4^,^5^. Any one of these or a combination of these phonetic characteristics in the present IDS stimuli may have generated the mismatch response due to their acoustic salience and heightened valence^73^ in IDS compared with ADS, but the heightened response to IDS may also be due to infants’ overall familiarity with the infant directed register^74^. More extensive exposure to this register in comparison to ADS may also lead to the preferential neural processing found here, whereby infants are more successful at detecting and attending to communicative information directly addressed to them in their speech input.

The results of this study conform with behavioural evidence that young infants successfully discriminate the infant-directed and adult-directed speech registers, and augments this, as the neural response patterns show a difference in the quality of responses to each register. Infants show a preference for IDS in behavioural paradigms, which has been attributed to the unique prosodic characteristics of this register such as heightened pitch, exaggerated pitch range, and positive affect^75^. These previous studies usually present infants with larger speech samples such as utterances or words, but the use of a single vowel exemplar from each register here was *also* sufficient to elicit differences in the neural responses to the two registers in infants and adults. We used vowels extracted from naturalistic IDS (i.e., speech produced in a live interaction with an infant) that contains all the characteristic acoustic and linguistic qualities of this register, which can be absent in computer-generated or acted-out speech^76^. However, it must be noted that the use of these naturally produced vowel exemplars does not allow us to determine whether a particular component of IDS was responsible for eliciting the mature MMN responses in infants or if a number of components were acting in unison. That is, it is possible that the mature MMN response is elicited by the greater acoustic salience of the IDS vowel over ADS vowel and that this greater salience is, in turn, due to a single acoustic quality and valence of IDS (e.g., greater format values) than its ADS counterpart, or a combination of qualities and valences (e.g., greater format values and heightened formant values). This is of particular interest given that while the acoustic, affective, and linguistic qualities of the stimuli in this study are typical of IDS, they are not unique to this register nor do they necessarily co-occur in all speech addressed to infants. It will be of interest in future research to investigate whether similar response patterns can be generated by vowels that were not produced in IDS, but that share some of the acoustic qualities characteristic of this register. In addition, these individual components can be absent or modified even in IDS, when speech is produced under certain circumstances, e.g., by mothers with post-natal depression^77^,^78^ or when IDS is addressed to infants with impaired hearing^15^,^79^. Thus, it is of interest for future research to focus on disentangling the role of each IDS quality on neural processing of speech and its implications for the development of early language skills in typically and atypically developing infant populations.

The lack of difference between the obligatory response to ADS and IDS in the infants here fails to confirm previous studies that have shown that IDS generated larger responses between 150–400 ms in infants using words^33^ and isolated vowels^34^. It must be noted, however, that the method employed in the present study was specifically optimised to elicit MMN responses, while the previous studies have mainly focused on eliciting obligatory responses. That is, in our study, an inter-stimulus interval (ISI) of 500 ms was used because shorter ISIs generate MMNs with high amplitude^73^. Previous studies focused on eliciting obligatory responses have used ISIs above 1 s; shorter ISIs have been shown to reduce the amplitude of the obligatory responses in infants due to neural refractoriness^80^. Hence it is possible that any small differences in the obligatory response between IDS and ADS were not detected here due to any such reduced amplitude. A second and related factor is the width of the analysis time window (epoch). Since the paradigm in the present study was optimised for the generation of MMN (500 ms ISI), the analysis window was between −100 and 400 ms. It is possible that a difference between ADS and IDS neural patterns emerges at a later time window in infants. Finally, the difference in findings could also be related to the differences in infant age. In this study 9- to 10-month-old infants were tested whereas previous studies tested 6- to 12-month-olds^33^ and 6- and 13month-old infants^34^. Since it is established that characteristics of IDS change in the first year of life^17^, it is possible that the neural response to IDS is also different at different ages. Interestingly the findings of the present study *are* similar to those of the 6 month olds in Zangl and Mills^33^ where ERPs to IDS and IDS did not differ in the 0 to 400 ms time range. Therefore, to better understand and enrich the novel finding of obligatory response to IDS and ADS found here, further studies are required across a variety of ages and with different ISIs.

In summary, this study examined cortical speech processing of ADS and IDS and their discrimination in infants and adults. Adults showed differences in early cortical processing between ADS and IDS as indexed by the obligatory responses. Speech discrimination, measured by MMN in adults did not differ between IDS and ADS whereas IDS generated greater attention shift as indexed by P3a in adults. In contrast, infants did not show a difference in cortical processing of ADS and IDS in their obligatory ERPs; instead, infants generated both positive MMR and negative MMN for IDS but only a positive MMR for ADS. The presence of an adult-like MMN for IDS deviants in infants is indicative of easier and more mature cortical speech discrimination for the style of speech specifically tailored for them (either by parental or infant design).

## Additional Information

**How to cite this article**: Peter, V. *et al.* Mature neural responses to Infant-Directed Speech but not Adult-Directed Speech in Pre-Verbal Infants. *Sci. Rep.* **6**, 34273; doi: 10.1038/srep34273 (2016).

## Supplementary Material

Supplementary Information

## Figures and Tables

**Table 1:** Acoustic properties of IDS and ADS stimuli.

	F0	Duration	F1	F2
ADS	146.19	103.99	541.48	1598.65
IDS	375.13	104.72	614.61	1622.12

**Table 2:** Significant clusters in the cluster permutation tests.

	Comparison	Cluster type	Time window	p
Adults	ADS Deviant-ADS Standard	Positive	68–195 ms	0.024
		Positive	209–272 ms	0.024
		Negative	67–192 ms	0.001
	IDS Deviant-IDS Standard	Positive	68–163 ms	0.001
		Positive	175–350 ms	0.022
		Negative	76–166 ms	0.001
		Negative	167–380 ms	0.010
Infants (0.1–20 Hz)	ADS Deviant-ADS Standard	Positive	92–380 ms	0.009
		Negative	120–358 ms	0.028
	IDS Deviant-IDS Standard	Positive	231–338 ms	0.019
		Negative	235–372 ms	0.036
Infants (3–18 Hz)	IDS Deviant-IDS Standard	Negative	153–219 ms	0.020
