# Supplementary material for: Mature neural responses to Infant-Directed Speech but not Adult-Directed Speech in Pre-Verbal Infants
Source: Sci Rep. 2016 Sep 28;6:34273. doi: 10.1038/srep34273 (PMC5039709; doi:10.1038/srep34273)
Supplement: Supplementary Information [file srep34273-s1.pdf]

Mature neural responses to Infant-Directed Speech but not Adult-Directed Speech in Pre-Verbal Infants

Varghese Peter, Marina Kalashnikova, Aimee Santos, Denis Burnham

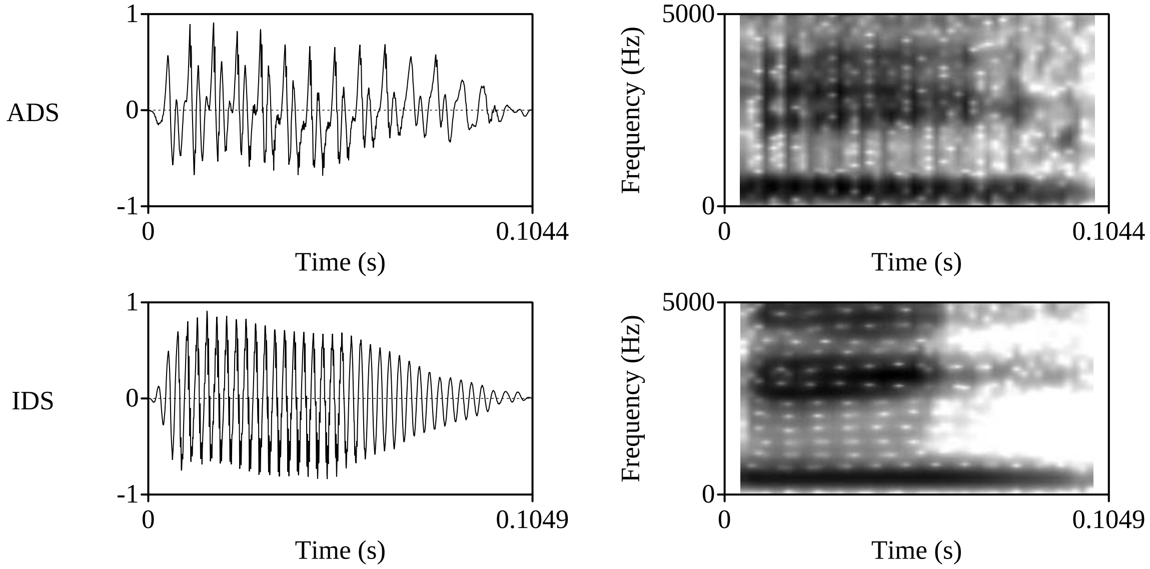

Experiment 1: ADULTS

MMN

Factor	F	p	Partial η^2
Stimulus	F(1,19)=0.02	0.879	0.001
Location	F(1.20,22.75)=11.03	0.002	0.367
Hemisphere	F(1,19)=2.13	0.160	0.101
Stimulus*Location	F(1.15, 21.89)=0.53	0.497	0.027
Stimulus*Hemisphere	F(1,19)=0.03	0.865	0.002
Location*Hemisphere	F(2,38)=1.21	0.311	0.060
Stimulus*Location*Hemisphere	F(1.44,27.29)=0.59	0.890	0.003

Main effect of location: MMN at frontal (M=-0.45, SE=0.11) and central (M=-0.33, SE=0.07) locations were negative where the response at occipital was positive (M=0.19, SE=0.08).

P3a

Factor	F	p	Partial η^2
Stimulus	F(1,19)=13.34	0.002	0.412
Location	F(1.32,25.16)=26.639	0.001	0.584
Hemisphere	F(1,19)=0.20	0.660	0.010
Stimulus*Location	F(1.18,22.45)=7.53	0.009	0.284
Stimulus*Hemisphere	F(1,19)=0.35	0.559	0.018
Location*Hemisphere	F(1.44,27.31)=0.30	0.743	0.016
Stimulus*Location*Hemisphere	F(1.48,28.19)=1.759	0.196	0.085

Main effect of stimulus: IDS generated larger P3a (M=0.21, SE=0.03) compared to ADS (M=0.08, SE=0.03).

Main effect of location: P3a at frontal (M=0.47, SE=0.09) and central (M=0.39, SE=0.06) locations were larger than occipital (M=-0.43, SE=0.09).

Stimulus*Location: Follow up one way ANOVA at each location

Factor	F	p	Partial η^2
Frontal	F(1,19)=13.90	0.001	0.422
Central	F(1,19)=3.43	0.083	0.150
Occipital	F(1,19)=2.80	0.111	0.129

Main effect of stimulus at frontal location: IDS generated larger P3a at frontal (M=0.70, SE=0.10) than ADS (M=0.24, SE=0.12).

Experiment 2: INFANTS

100-200 ms

**Factor**	**F**	**p**	**Partial η^2**
Stimulus	F(1,19)=0.55	0.471	0.035
Location	F(1.34,20.15)=5.08	0.027	0.253
Hemisphere	F(1,19)=0.01	0.997	0.001
Stimulus*Location	F(1.17, 17.59)=0.21	0.692	0.014
Stimulus*Hemisphere	F(1,19)=0.49	0.493	0.032
Location*Hemisphere	F(1.26,18.90)=0.12	0.789	0.008
Stimulus*Location*Hemisphere	F(1.44,27.29)=1.11	0.342	0.069

Main effect of location: Pairwise comparisons showed no significant differences across conditions in the 100 to 200 ms window.

200-300 ms

**Factor**	**F**	**p**	**Partial η^2**
Stimulus	F(1,19)=0.09	0.773	0.006
Location	F(1.27,19.02)=11.38	0.002	0.431
Hemisphere	F(1,19)=1.26	0.280	0.077
Stimulus*Location	F(1.24, 18.65)=0.12	0.947	0.001
Stimulus*Hemisphere	F(1,19)=0.07	0.789	0.005
Location*Hemisphere	F(1.20,18.05)=0.05	0.866	0.003
Stimulus*Location*Hemisphere	F(1.44,27.29)=0.41	0.669	0.026

Main effect of location: ERP responses at frontal (M=1.05, SE=0.34) and central (M=0.57, SE=0.27) locations was positive which was significantly different from the occipital response which was negative (M=-1.35, SE=0.36).

300-400 ms

**Factor**	**F**	**p**	**Partial η^2**
Stimulus	F(1,19)=0.99	0.336	0.062
Location	F(1.36,2034)=2.73	0.105	0.154
Hemisphere	F(1,19)=0.52	0.484	0.033
Stimulus*Location	F(1.37, 20.47)=0.28	0.676	0.018
Stimulus*Hemisphere	F(1,19)=0.04	0.838	0.003
Location*Hemisphere	F(1.36,20.47)=0.19	0.747	0.012
Stimulus*Location*Hemisphere	F(1.44,27.29)=0.77	0.471	0.049
